# Rapid encoding of an internal model for imitative learning

**DOI:** 10.1098/rspb.2013.2630

**Published:** 2014-04-22

**Authors:** Mugdha Deshpande, Fakhriddin Pirlepesov, Thierry Lints

**Affiliations:** Department of Biology, Texas A&M University, College Station, TX 77843, USA

**Keywords:** imitation, vocal learning, songbird, zebra finch

## Abstract

As in human infant speech development, vocal imitation in songbirds involves sensory acquisition and memorization of adult-produced vocal signals, followed by a protracted phase of vocal motor practice. The internal model of adult tutor song in the juvenile male brain, termed ‘the template’, is central to the vocal imitation process. However, even the most fundamental aspects of the template, such as when, where and how it is encoded in the brain, remain poorly understood. A major impediment to progress is that current studies of songbird vocal learning use protracted tutoring over days, weeks or months, complicating dissection of the template encoding process. Here, we take the key step of tightly constraining the timing of template acquisition. We show that, in the zebra finch, template encoding can be time locked to, on average, a 2 h period of juvenile life and based on just 75 s of cumulative tutor song exposure. Crucially, we find that vocal changes occurring on the day of training correlate with eventual imitative success. This paradigm will lead to insights on how the template is instantiated in the songbird brain, with general implications for deciphering how internal models are formed to guide learning of complex social behaviours.

## 1. Introduction

Imitative learning enables individuals to acquire behaviours essential to survival and fitness by observing the activities of conspecifics. Songbirds provide a facile experimental model for studying imitation in the context of vocal learning and represent the best-understood animal model for human speech and language development [1,2]. Young male zebra finches begin to produce soft, babbling-like vocalizations, termed subsong, at around 30 days post hatch (dph). The essential first step in the vocal imitation process is formation of a template (the memory of the tutor song) that will guide the course of vocal development. During subsequent vocal ontogeny, the auditory feedback of the bird's own song is compared with the stored template to generate a local error signal which is progressively reduced through gradual modifications to vocal output. This process results in a mature crystallized song that closely resembles the tutor song model by 90 dph [3]. In the absence of any exposure to adult tutor song, young males develop isolate songs that are less structured and contain atypical syllables [4].

Exposing song-naive juveniles to adult songs through an operant training paradigm provides a way to compare learning trajectories across birds under rigorously controlled conditions [5]. Moreover, by operant tutoring with a select few adult songs, it is also possible to assess the efficacy of vocal learning under experimental conditions used by different laboratories. Continued training from 35 dph, with access to the song model restricted to two sessions daily (amounting to 40 motifs, or 30 s of song per day) for two months, can induce a close imitation of the tutor song, whereas high song model abundance (for example, several thousand tutor song motifs per day) severely reduces imitation accuracy [5]. Abbreviated operant training with the same tutor song, but lasting only 2 days, also results in reduced imitation [6]. However, even under the latter conditions, the timescale of training is not ideally suited to dissecting mechanisms of template encoding and a narrower timeframe would be preferable. Our goal was therefore to determine whether juvenile male zebra finches were capable of rapid and detailed template formation in an even more restricted operant paradigm. We have examined this possibility using the same tutor song model used in protracted operant training experiments, which thereby provide a baseline of ‘normative’ vocal ontogeny data [5,7,8].

Whether zebra finches possess an aptitude for rapid and detailed template formation within a matter of hours has not been adequately addressed. Nevertheless, from a comparative ethological perspective, other songbirds are capable of song imitation based on limited learning opportunities. Song sparrows (*Melospiza melodia*) [9] and European blackbirds (*Turdus merula*; cited in reference [9]) imitate species-specific adult song presented on a single day. Spectacularly, nightingales (*Luscinia megarhynchos*) are capable of imitating multiple songs heard only a few times [10]. These observations span half a century, but have not ignited study of the neural basis of template encoding in these species; indeed, the vast majority of song system neuroscience remains focused on the zebra finch.

In zebra finches, juvenile males typically exhibit rapid vocal changes on the second day of training [7] and by the fourth training day, prototype sounds are produced that can be identified with syllables of the final crystallized imitation [11]. Although these data indicate that aspects of the song model are acquired within the first few days of training, they do not reveal whether the template is finely wrought at the earliest stages, or a rough guide that is updated by subsequent training opportunities. To address this issue, we sought to time lock template acquisition by limiting the opportunity to acquire the song model to a single session of operant training and examined whether this restricted exposure can induce imitation of the song model.

We show that, in zebra finches, a single episode of tutoring can exert lasting effects on song development. Total tutor song exposure of 75 s or less, distributed on average over a 2 h training session, was sufficient to initiate the song imitation process in juvenile males in an age-dependent fashion. As this training session represents their first and only encounter with an adult male song, we studied the initial vocal response of young male zebra finches to this highly salient event. In a subset of birds, rapid vocal changes were observed within hours and, in some cases occurred during the training session itself. Moreover, changes to the acoustic features of subsong on the day of training correlate with imitative similarity achieved by adulthood. Thus, initial encoding and/or consolidation of the song template may not be dependent on night sleep. Our behavioural findings establish a useful predictive framework for future studies: in the ongoing search for the template, an informative electrophysiological signature exhibited by neurons encoding the tutor song memory would be that they (i) exhibit tutor song-evoked neural activity that evolves over the course of the first and only training session, and (ii) that these changes in neural activity correlate with training-day vocal change and final imitative success.

## 2. Methods

All animal procedures were approved by the Texas A&M IACUC committee. In total, 89 zebra finch males were used for training experiments as described below (electronic supplementary material, table S1). Nestlings were removed from the breeding colony along with their mothers at 7 dph and were raised without any further exposure to adult males. Fledglings were weighed, banded and sex-typed at 30 dph. Juvenile males were moved to sound attenuation boxes between 32 and 37 dph, from which time onwards their songs were recorded continuously. All sound recording and training experiments were conducted using Sound Analysis Pro (SAP) software [12], unless specified otherwise. The birds remained acoustically isolated throughout the song development period until 90–100 dph. Ten birds were used as untrained controls and acoustically isolated in a similar fashion, but not exposed to an adult tutor song model.

### (a) Training set-up

For the initial operant training experiments, 33 males were trained, at specific ages, with audio playbacks of an adult male zebra finch song model ‘Samba’ (duration of single motif = 750 ms; figure 2*a*). The operant training set-up was slightly modified from Tchernichovski *et al.* [5]. A plastic model of an adult male zebra finch was attached to a perch and placed near one end of the cage, in front of an acoustic speaker. A plastic model of a female zebra finch was placed at the opposite end of the cage. After a minimum of 2 days of baseline recordings of subsong were obtained, a string was attached to the beak of the female model. Pulling of the string activated a key (Cherry Electrical E22-85HX). This ‘key-peck’ resulted in adult song playback. The amplitude of song playbacks was adjusted to approximately 75 dB, as measured at the opposite end of the cage (dimensions in cm 43.5(L) × 26(W) × 30.5(H)) from the speaker. The number of motifs played per playback was randomized between 2 and 8 (that is 1.5–6 s of song playback). A training session could commence any time between 09.00 and 17.00 on a single day at the required age of the bird (35, 45 or 60 dph) and was limited to 20 key-pecks (i.e. approximately 100 motifs or 75 s of song playback). By way of contrast, the same song model, Samba, has been used in an operant training paradigm wherein juvenile males were allowed two training sessions daily (for a maximum of 40 motifs per day), throughout the vocal development process (operant training typically spanning from 42 to 90 dph) [5,7,8]. In the current experiments, all birds included for data analysis received between 15 and 20 key-pecks on the sole day of training; 90% (30/33) achieving the full quota. Average duration to finish the quota of 20 key-pecks was 2 : 10 h (±1 : 46 h; total range: 0:14:46–5:53:37 h). Neither the duration of the training session (time between first and last key-peck) nor the timing when the session was initiated during the day affected the final imitative outcome (electronic supplementary material, figure S1).

For audio-visual training experiments, 22 males were trained at 35 dph and 24 males were trained at 45 dph (*N* = 46 total). The song model videos were created by recording female-directed song of an adult male (m210). Individual high-definition (720p) video clips containing two to eight motifs of singing were created and Apple iPads were used to deliver the stimuli through a *keynote* presentation. Pulling on a string attached to a key resulted in a playback from the iPad in a similar manner to the operant paradigm as described above. The key (Cherry Electrical E22-85HX) was wired to the iPad via a penny cemented with Cotronics Duralco 120 conductive epoxy onto a screen protector covering the iPad capacitive screen. The stimulus files for simultaneous audio-visual playbacks were modified to generate audio-only and staggered audio-visual stimuli. For audio preceding video (AV) and video preceding audio (VA) stimuli, a gap of 2 s (silence, black screen) was inserted between the offset of the first stimulus modality and the onset of the second stimulus modality. For most experiments, birds were also monitored and recorded using a security camera system to follow their behaviour during key-peck events.

### (b) Data analysis

Audio data obtained from SAP was first processed using custom-written Matlab codes to separate sound files containing songs from those with cage noises and calls. Briefly, spectrogram images were created from the sound files and visually inspected for song. Sound files containing song were moved to a new folder and band-pass filtered (400 Hz–18 kHz) by another custom-written Matlab code.

#### (i) Probability distribution function and principal components analysis

For all birds, 30 song files containing a single motif each were selected from the crystallized song recordings obtained from birds at 88 dph or older. Spectral features for 10 ms windows of sound (raw features) were obtained from these files using SAP. The following features were used in the probability density function (PDF) analysis. Wiener entropy (WE) is a measure of noisiness in the acoustic data (white noise has WE = 1, and pure tone has WE = 0), and entropy variance (EV) is the variance in the WE per bin. Frequency modulation (FM) reflects the degree of angular slope in the frequency traces. Goodness of pitch (GP) measures the periodic nature of harmonic sound [13].

For constructing the principal components analysis (PCA), cumulative distribution functions (CDFs) of song raw feature values for the song files described above were plotted in Matlab by dividing the data into 100 bins. The final PCA was created by using the cumulative frequency in each bin for specific features as variables. For the juvenile subsong PCA, data from subsongs at 42–44 dph for six untrained birds and six birds randomly chosen from those destined to be trained at 45 dph were pooled.

#### (ii) Similarity scores

For each bird, 30 examples of crystallized song were selected and their amplitude envelopes normalized to the song model file. Similarity scores were generated by the corresponding module in SAP using ‘asymmetric’ and ‘time course’ mode. The window for scoring was increased to 25 ms, and scores were calculated at probability, *p* = 0.05. This software generates the measures for similarity based on the Euclidian distances across different spectral features (pitch, WE, GP, FM, amplitude modulation) between the two songs [12]. The similarity score used here is a combined score that takes into account per cent similarity, accuracy and sequential match and is referred to as the ‘cumulative similarity score’. This scoring approach sets a higher bar than that based on acoustic similarity alone (i.e. without temporal/sequence specificity included). The spectrograms in figure 2*a* and electronic supplementary material, figure S3 illustrate this point. Here, cumulative similarity scores are used throughout, but both scoring approaches yielded essentially identical statistical results in the between-group comparisons presented in this report.

#### (iii) Song features analysis

Syllable tables for song files were created using SAP. Mean values and standard deviations for the feature values were calculated using a custom-written Matlab code. All statistical analysis was performed using Origin Pro.

## 3. Results

### (a) Sustained vocal changes are induced by a single training session

**Figure 1.**
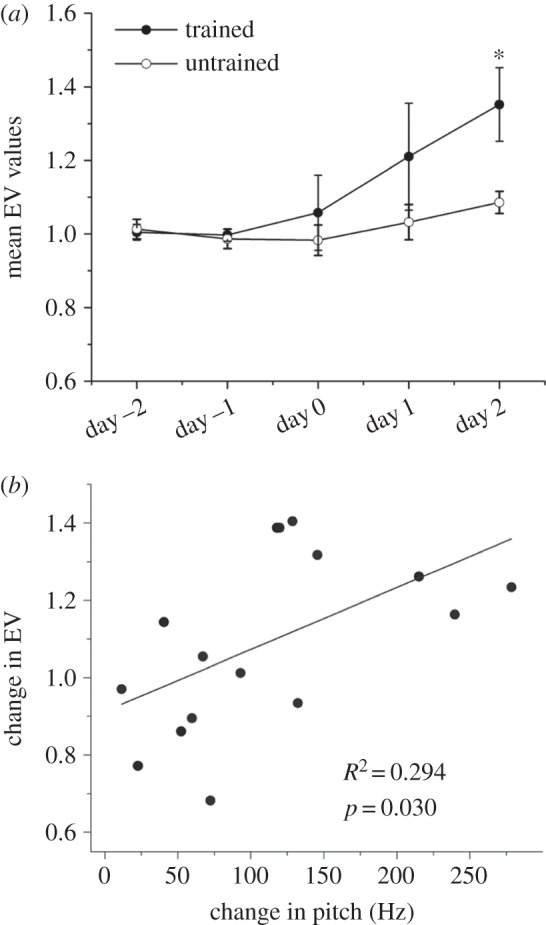
Sustained vocal changes are induced by a single training session*.* Song features were analysed from the pooled data of birds trained at 35 dph (*N* = 8) and 45 dph (*N* = 9) for a 5 day window centred on the training day (day 0; filled circles) and untrained birds between ages 40 ± 2 and 45 ± 2 dph (open circles; *N* = 8). (*a*) Mean EV values normalized by dividing mean EV for each day with baseline EV calculated as the average of EV values for 2 days prior to training (day −2, −1). Error-bars denote the standard error of the mean. Asterisk; mean EV for the second post-training day is significantly higher than the baseline for trained birds (post hoc Tukey's test, baseline versus day 2, *p* = 0.01; day 0 versus day 2, *p* = 0.03) as well as age-matched untrained birds (two sample *t*-test, *p* = 0.024). (*b*) Fold change in mean EV values over day 0, 1 and 2 correlates with the change in mean pitch (*R*^2^ = 0.294, *F* = 5.7, *p* = 0.03). For graphical clarity, one outlier with exceptional change in EV (2.55) and pitch (448 Hz) was omitted (when included, *R*^2^ = 0.64, *F* = 30.3, *p* = 6.0 × 10^−5^).

**Figure 2.**
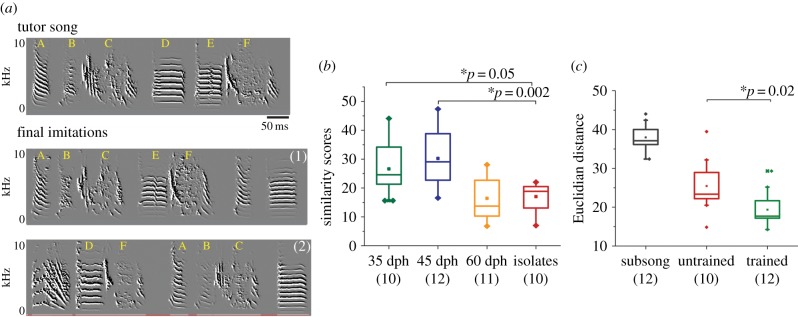
A single training session can lead to commitment to the tutor song model. (*a*) Spectrographs demonstrating song imitation following SST at 45 dph, for two birds capturing many features of the song model, albeit with syllabic rearrangement. The per cent similarity between the tutor song and songs 1 and 2 is 79.7 and 78.8, respectively. Cumulative similarity scores (imitation accuracy and temporal sequence in addition to similarity) for birds 1 and 2 are 41.4 and 47.4, respectively. Mean pitch of syllable D in the tutor song is 718 and syllable E is 635 Hz. The syllable marked as a copy of D in the imitation by bird no. 2, has a pitch of 728 Hz and therefore is closer to D in feature properties, despite being positioned adjacent to F. Additional exemplars spanning the range of ‘Samba’ imitation are provided in electronic supplementary material, figure S4. (*b*) Birds trained at 35 and 45 dph show significantly higher similarity to the song model when compared with the untrained controls (post hoc Tukey's test; d45 versus isolates *p* = 0.002, d35 versus isolates *p* = 0.05). (*c*) Comparing Euclidian distances across all PCs shows trained birds are significantly closer to the model than untrained birds (a Euclidean distance of zero denotes identity to the tutor song model; one-way ANOVA, *F* = 44.4, *p* = 7.8 × 10^−10^). For (*b*) and (*c*), each box outline denotes 25th–75th percentile with the midline denoting the median, mean is shown by filled square and outliers are marked by filled diamonds. The numbers in brackets indicate sample size (*N*).

We first tested whether typical initial trajectories of vocal change can be induced by a single (audio-only) operant training session (single session training, SST) and maintained in the absence of any subsequent tutor song exposure. Juvenile males (*N* = 33), raised out of earshot of adult song, were placed singly into sound isolation boxes from 30 dph in order to record juvenile subsong and establish a baseline for the song features analysis. We then examined the vocal output of juvenile males trained with song model Samba during and following a single training session at 35 dph (*N* = 10) or 45 dph (*N* = 12), in order to track the changes induced by exposure to the adult song. Two birds trained at 35 dph and three birds trained at 45 dph were omitted from this analysis owing to lack of singing activity on more than 2 days before or after training, giving *N* = 17 birds for which data are presented in figure 1 (35 and 45 dph combined; these groups are not statistically different in their imitative response to SST, as shown in figure 2*b*). We used mean EV as a measure of syllable structure [3]. We observed a progressive increase in the mean EV on the days following training, even in the absence of subsequent training, with significant gain in mean EV values (higher by 29.4%) across the combined 35 and 45 dph groups on the second post-training day (figure 1*a*; repeated-measures ANOVA, *F*_3,30_ = 4.43, *p* < 0.05; post hoc Tukey's test, baseline (average of mean EV on days −1 and −2) versus day 2, *p* = 0.01; day 0 versus day 2, *p* = 0.03). For isolate birds over the same age range, there was a slight but non-significant trend to increased mean EV values across a similar 5 day interval (figure 1*a*; no significant differences in any between day comparison using repeated-measures ANOVA), possibly reflecting gradual maturational changes in the song system. Moreover, in 10 of 17 trained birds, mean syllable pitch increased by 15% or more during the first 2 days following training (one-way ANOVA, *p* = 0.06, Tukey's test, baseline (average of mean syllable pitch on days −1 and −2) versus day 2, *p* = 0.04), whereas over the same duration 0 of 10 age-matched isolates changed pitch to the same extent (one-way ANOVA, *p* = 0.97, Tukey's test, baseline versus day 2, *p* = 0.99). These changes in pitch and EV within the first few days after training were positively correlated (figure 1*b*; Pearson's *R* = 0.54, *F* = 5.7, *p* = 0.03). Importantly, these data demonstrate that SST is sufficient to mount and sustain experience-dependent ontogenetic vocal change in the absence of any further adult tutor song exposure.

### (b) A single training session can lead to commitment to the song model in juvenile zebra finches

The earliest post-training changes to subsong may be biased by the acoustic characteristics of the tutor song [14], but might not necessarily reflect ultimate commitment to the tutor song model. To test the long-term effect of this restricted song exposure paradigm, we maintained SST birds in acoustic isolation until 90 dph. We measured the imitative success of SST birds using similarity scores that take into account overall similarity, local accuracy and sequence specificity with respect to the tutor song. The songs of individual birds showing high similarity scores capture many details of the song model (figure 2*a*). Similarity scores of adult songs (recorded at 88–100 dph) from birds trained at 45 dph (mean = 30.2 ± 9.5, *N* = 12) and 35 dph (mean = 26.6 ± 9.4, *N* = 10) were significantly higher than those produced by untrained birds (mean = 17.03 ± 4.7, *N* = 10), which indicate the baseline score owing to chance (figure 2*b*; post hoc Tukey's test; d45 versus isolates *p* = 0.002, d35 versus isolates *p* = 0.05, d35 versus d45 *p* = 0.719). Note that higher scores are obtained when based on spectral similarity alone, ignoring temporal structure (e.g. 45 dph trained mean = 52.8 ± 4.5; untrained 29.5 ± 2.7, *p* = 0.0007).

Training of birds at 60 dph did not induce appreciable imitation under SST conditions (mean = 16.4 ± 7.14, *N* = 11; post hoc Tukey's test d60 versus isolates *p* = 0.998). This decline in the ability to imitate the song model suggests that, although raised in isolation from adult male song (thereby potentially extending the sensory phase of learning), by 60 dph the critical period for acquisition of a song model accessible to imitative learning mechanisms may be drawing to a close. This accords with changes in song system neural plasticity occurring in isolate reared birds around this age [15]. Despite the very different life experience of 60 dph SST birds and juvenile males exposed to successive live adult male tutors throughout vocal ontogeny [16], the sensory phase for acquisition of a viable song template may in either case close by around 60 dph.

Within each age group (and also when 35 and 45 dph group data are combined; not shown), there was no relationship between final imitation success and the duration of training, ruling out massed versus spaced training effects (electronic supplementary material, figure S1*a*–*c*). There also was no appreciable circadian effect on template acquisition, as the timing of training onset during the day did not correlate with final song similarity score (electronic supplementary material, figure S1*d*–*f*). Therefore, we conclude that the major source of individual variation in the success of imitation under SST conditions is likely due to intrinsic, rather than due to experiential, differences between birds.

To more inclusively capture changes owing to SST across the full range of imitation, without segmenting songs into syllables, we used the distribution of spectral features such as WE, FM and GP [6]. PDF plots for initial raw feature values of pre-training subsong for the SST group and untrained group birds were similar to each other (*N* = 6/group; electronic supplementary material, figure S2*a*–*c*). We then compared the distribution of raw feature values in adult songs of all birds trained at 45 dph (*N* = 12) and untrained birds (*N* = 10). By 90 dph, PDFs for WE of adult songs of SST birds were significantly closer to the PDF of tutor song WE than were untrained bird PDFs (electronic supplementary material, figure S2*f*). PCA of the raw features data also showed an approach towards the tutor song model by SST birds (electronic supplementary material, figure S2*g*) [17]. We then assessed the effect of training on the Euclidian distance for each trained birds’ song from the song model across all PCs. The distance from the song model was significantly closer for adult songs of the trained birds when compared with those of the untrained adult birds (figure 2*c*; two-sample *t*-test, *p* = 0.02), reinforcing our earlier conclusion that SST biases the song development of younger birds towards acoustic features of the tutor song model.

Nevertheless, adult songs of *both* trained and untrained groups show closer Euclidean proximity to the tutor song model than do untutored subsongs (one-way ANOVA, *F* = 44.4, *p* = 7.8 × 10^−10^). In principle, the closeness of tutored songs to the song model could be due to a general enhancement of age-dependent maturation of song development, rather than imitation of the model song, *per se*. To address this possibility, we calculated the Euclidian distance of SST birds’ songs to 10 randomly selected wild-type, adult non-tutor songs (electronic supplementary material, table S2). Inclusion of these 10 non-tutor birds in PCA along with Samba and 45 dph Samba-trained birds (electronic supplementary material, figure S2*h*) reveals distinct spatial distributions of Samba-trained and non-tutor songs. Moreover, in this PCA, the songs of five Samba-trained birds were closer to Samba than were the songs of any normally reared, non-tutor male. We then compared the distances between trained birds’ songs and the song model, relative to their distances from other adult songs. Of these 10 comparisons (paired *t*-test), five showed that trained birds’ songs were significantly closer to the song model than other adult songs and four showed no significant difference between the distances of trained birds’ songs from the song model compared with their distance from other adult song. Significantly, however, the adult songs showing proximity to the trained birds’ songs equal to that of the model were also significantly closer to the song model itself. Moreover, the single adult non-tutor song that trained birds were more similar in Euclidean distance to than Samba, was in fact the most similar to Samba among all the adult songs selected (electronic supplementary material, table S2). To further assess whether specific features of the song model are acquired by SST training, we also compared song similarity scores of 45 dph SST Samba birds with adult non-tutor songs. We selected one song (m495pi) that showed significantly higher Euclidian distance from the trained birds’ songs than Samba and another (mD10092) where the Euclidian distance from the trained birds songs was not significantly different from their distance to Samba (electronic supplementary material, figure S2i). Similarity scores of trained birds to Samba were significantly greater than to either non-tutor song (repeated-measures ANOVA; electronic supplementary material, figure S2j). Moreover, on an individual basis, 10 of these 12 birds showed higher similarity to Samba than they did to m495pi, and all 12 birds showed higher similarity than they did to D10092. Remarkably, taken together, our data demonstrate that a single training session comprised of less than or equal to 75 s of song playback, constrained within 2 h (on average), is sufficient for template encoding and the diversion of vocal ontogeny towards song features specific to the tutor song model.

**Figure 3.**
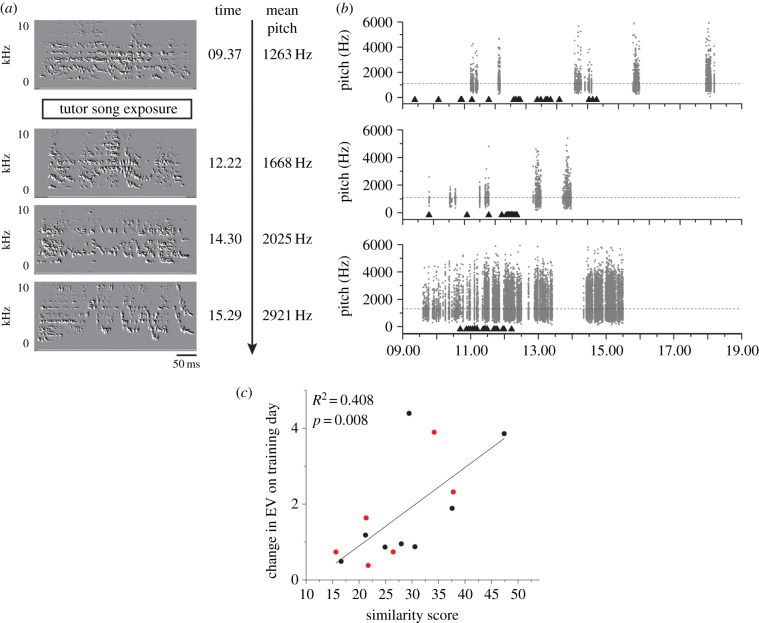
Vocal changes on the day of training correlate with final imitation. (*a*) Spectrographs showing an example dramatic increase in subsong pitch on the day of training at 45 dph. First playback occurred at 09.41. (*b*) Three examples of pitch elevation on the day of training with respect to the timing of operant-triggered tutor song playbacks. The top panel shows data for a bird trained at 35 dph and the middle and bottom panels show 45 dph training day vocal trajectories. Each point indicates mean pitch for a syllable; the black triangles indicate the timing of a tutor song playback and the dashed line indicates the average pitch for the 2 days preceding training. (*c*) Birds trained at 35 (red dots) and 45 dph (black dots) show significant correlation between their final similarity score and the fold change in 95 percentile entropy variance (EV) values on the day of training (*R*^2^ = 0.408, *F* = 9.96, *p* = 0.0082). *N* = 14.

We next investigated whether song features change on the training day itself, or whether a night sleep is required before such changes are manifest. Changes in the pre-motor pathway for song production occur during the first post-training night sleep and include the emergence of robust high-frequency burst firing of RA (robust nucleus of the arcopallium) neurons and the enlargement and stabilization of dendritic spines in another key song control nucleus, HVC (not an acronym) [14,15]. Examining pitch and EV on the day of training for the combined group of birds trained at 35 or 45 dph we found that, for six of 17 birds, the syllable feature values changed more than 2 s.d. from the average amount of daily change observed across this group in the two pre-training days. Vocal changes occurred within hours of the onset of training and sometimes even before the training session closed (figure 3*a,b*). Together with another report [15], our data demonstrate that night-sleep-dependent processes are not a necessary precondition for the first expression of imitative vocal change, as has been proposed [14]. It will be of interest to further identify alterations in RA and HVC activity that occur in the awake state (or during daytime naps) to prefigure vocal change, as opposed to those that are specific to night sleep, consolidating vocal changes already set in motion.

Significantly, we found that the final similarity score of adult songs of birds trained at 35 or 45 dph positively correlated with the degree of change in 95th percentile EV values on the day of training (Pearson's *R* = 0.67, *p* = 0.008, *N* = 14; figure 3*c*). Ninety-fifth percentile EV values have been used as a measure for the most structured song elements being produced in the given time-window [14]. We tested whether the varying levels of song production for individual birds on the training day affect the final similarity score or the degree of change in 95th percentile EV values on the day of training. We calculated the total number of song syllables produced by each bird on the training day as a measure for total song production. There were no statistically significant correlations either between the amount of singing on the day of training and final imitation score (Pearson's *R* = −0.12937, *p* = 0.62), or between the amount of singing and 95th percentile changes in EV (Pearson's *R* = 0.45617, *p* = 0.1). We also found no evidence for any correlation between acoustic features of subsong produced prior to training (for example, mean pitch, mean EV, mean 95th percentile EV) and final imitative outcome (not shown). These results suggest that changes in top EV values on the training day can be used to predict final imitative success of crystallized adult songs in the absence of further tutoring opportunities.

### (c) Age-dependent changes in the effect of visual input on sensory acquisition of the song model

Constraining tutor song playback to a single session provides a very impoverished learning opportunity. As visual input facilitates speech and language learning in human infants, we tested whether augmenting auditory input with a visual stimulus during training might improve imitation success [18]. To assess whether naturalistic visual stimuli might be beneficial to the sensory acquisition of the tutor song, juvenile males were allowed to trigger operant playbacks of pre-recorded audio-visual clips of an adult male (m210) singing female-directed song to a silent off-camera female. The similarity between the song model and the final songs of birds given a single-session training at 35 and 45 dph in each of four groups was then compared. The audio-visual components of directed song were played either simultaneously (Sim) or in a staggered fashion where birds received either audio before video (AV) or vice versa (VA). In the fourth group, birds received audio-only operant playbacks of the tutor song.

**Figure 4.**
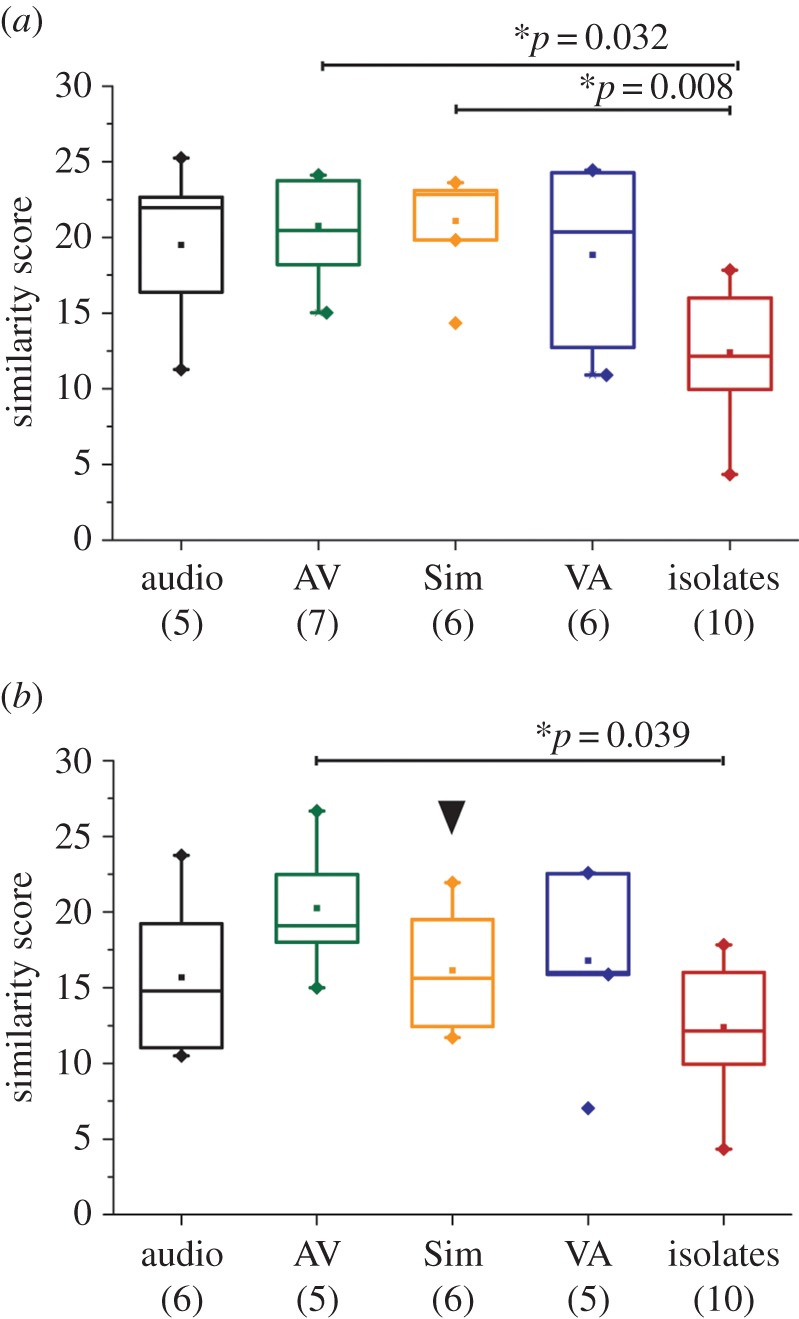
Sequential auditory–visual presentation of the tutor facilitates song learning at 35 dph. Boxplots showing average similarity scores for birds trained with SST using different paradigms of audio-visual playbacks at 45 (*a*) or 35 dph (*b*). Boxplot design is as described in figure 2. *p*-Values denote statistically significant differences between two groups, determined using one-way ANOVA followed by post hoc Tukey's test. Learning of the tutor song at 45 dph is robust to the parameters of audio-visual training used here. By contrast, at 35 dph, only the AV condition facilitates learning of this song model when compared with isolates.

When trained at 45 dph, AV and Sim groups showed significantly higher similarity to the song model than did the untrained group (one-way ANOVA; *F* = 4.8, *p* = 0.004: post hoc Tukey's test (comparison with untrained group); audio: *N* = 5, *p* = 0.061; AV: *N* = 7, *p* = 0.032; Sim: *N* = 6, *p* = 0.008; VA: *N* = 6, *p* = 0.076). However, there were no significant differences between the different groups of trained birds. By contrast, when trained at 35 dph, differences in the efficacy of these audio-visual training stimuli became apparent. Surprisingly, a single training session at this age was effective only under the AV condition (figure 4*b*; one-way ANOVA; *F* = 4.8, *p* = 0.004: post hoc Tukey's test (comparison with untrained group); audio: *N* = 6, *p* = 0.66; AV: *N* = 5, *p* = 0.039; Sim: *N* = 6, *p* = 0.55; VA: *N* = 5, *p* = 0.45). Moreover, birds trained at 35 dph with simultaneous audio-visual playbacks show significantly lower scores when compared with birds trained at 45 dph (two sample *t*-test, *p* = 0.004). It is noteworthy that similarity scores of the audio-only group trained to m210 are lower than birds trained to Samba, conceivably owing to the more complex nature of the m210 model (10 syllables when compared with six syllables). This increased tutor song complexity might also contribute to the trend towards poorer imitation in 35 dph birds trained with audio playbacks of m210 compared with 45 dph birds (a trend not seen in the average similarity scores of Samba-trained birds). However, it should also be noted that per cent similarity scores parallel the duration of a juvenile's imitation of tutor song [12], and so the longer duration (1290 versus 750 ms) and greater number of syllables in the m210 song than Samba will consequentially lead to lower similarity scores against m210, even if the same total duration of tutor sounds is copied by birds exposed to either song model.

## 4. Discussion

By winnowing the operant song training available to laboratory reared juvenile male zebra finches, we have demonstrated that it is possible to restrict the timing of template acquisition to just a few hours of juvenile experience (2.10 h ± 1.46 h). Moreover, a correlation between the gain in syllable EV on the day of training and the ultimate similarity of the juvenile's crystallized song to that of the song model was detected. This time-locking of the onset of the vocal imitation process and the rapid vocal change that ensue, now make it feasible to investigate the strength of song template encoding molecularly and electrophysiologically, within the first few hours of tutor song exposure. These results represent a valuable step towards elucidating the nature of the song template.

The variable learning exhibited by zebra finches (especially in response to SST) differs markedly from the uniformity of vocal learning in song sparrows under minimal training conditions [9]. In tape-tutored juvenile male song sparrows, reared without auditory experience of adult male song and then trained with a combination of heterospecific and conspecific playbacks at 50 dph, a select few tutored conspecific song types are copied very faithfully, irrespective of song model abundance. This contrasts with juvenile male zebra finch sensitivities to model abundance [5], perhaps indicating that in some respects hand-reared song sparrows trained at 50 dph may be more canalized in their ontogenetic vocal trajectories than 35–45 dph zebra finches. Indeed, when comparing the impact of early deafening, or isolation from conspecifics, on song development of closely related song and swamp sparrows (*M. melodia* and *M. georgiana*, respectively), many species-specific features of natural song are preserved despite experimental manipulation [19].

The differential responsiveness of juvenile male zebra finches and song sparrows to minimal tutoring might, for example, arise if the auditory system of juvenile male song sparrows were less dependent on exogenous song experience for maturation and therefore better prepared to capitalize on transient adult conspecific song input. Little is known about song sparrow auditory system maturation; however, in juvenile male zebra finches (but not in females), the absence of adult song exposure delays and/or impairs the development of auditory selectivity [20]. Isolate zebra finch males do not show stimulus-specific response patterns in adulthood as measured by event-related potential or functional magnetic resonance imaging studies. However, males that learn a model song through continuous operant training over the sensorimotor period show appropriate stimulus-specific auditory responses as adults [20]. A hypothesis to explain these differences proposes that maturation of stimulus specificity in male zebra finches is delayed to allow for plasticity during vocal learning [20]. Males trained with our minimal training paradigm show sensorimotor learning guided by a song model similar to continuous trained or colony reared birds in this study. However, save the single session of training, they are arguably closer to the isolate group in terms of their overall auditory exposure to adult conspecific song. Single-session operant training may represent an interesting strategy to assess relative contributions of auditory experience versus sensorimotor learning on shaping the stimulus specificity of auditory responses in juvenile male zebra finches.

Given that maturation of the juvenile male zebra finch auditory system is delayed relative to females, and that isolation from an appropriate song model during early vocal development is known to result in an extension of the sensory phase for song learning, it might be expected that SST birds trained at 60 dph would produce better imitations than they do. However, 45 and 60 dph isolate birds differ in multiple respects and very abbreviated training, such as the single-session training used here, may be especially susceptible to the diminishing neural plasticity underlying closure of the sensory acquisition phase of vocal learning. By 60 dph, many isolate birds begin to exhibit adult-like levels of reduced dendritic spine plasticity in HVC [15] and exhibit night-sleep-dependent song structure deterioration in response to tutoring that occurs with several days lag relative to the response of birds trained from 43 dph [3]. Conceivably, better imitation in birds given training from around 60 dph might depend on whether the tutor song model (or a short-lived memory trace of that song) remains available to the bird during the period of post-sleep deterioration that occurs several days after the onset of training. As yet, we do not know whether SST is sufficient to induce such delayed vocal plasticity in 60 dph + birds.

The audio-visual training set-up described here could provide a basis for novel strategies to explore neuroanatomical correlates of template storage. It remains to be tested whether embedding tutor song playbacks on a noisy background (e.g. emulating a natural colonial setting) can reveal intersensory facilitation under Sim conditions [21]. In this respect, some experimental advantage may pertain to the finding that birds trained at 35 dph imitate the song model better when given AV stimuli than when trained under VA or Sim conditions. This result is reminiscent of the Colavita visual dominance effect [22,23] whereby visual perceptual demands trump auditory perception, except when the auditory stimulus is presented ahead of the visual. There are still uncertainties about the basis for this phenomenon, however [22]. Further work is required in order to explore the applicability of the Colavita effect to studies of song learning. Nevertheless, the difference in song learning exhibited by 35 dph AV and Sim groups that nominally receive the same sensory input (at least in aggregate) might potentially be exploited in neural activity mapping studies to identify brain regions that contribute to encoding of the template.

Not surprisingly, the training paradigm described here results in less robust imitation than protracted operant or live tutor training. Mean per cent similarity for live tutored or operant tutored birds given prolonged training to the Samba adult tutor song model fall in the 60–80% range [5,8]. It is striking, however, that a minority of SST birds imitate similarly well and seem capable of forming an almost eidetic syllabic memory of the tutor song based on just 75 cumulative seconds of tutor song exposure (e.g. figure 2*a*). Although most SST birds develop poorer imitations (see electronic supplementary material, figure S4), the individual variability observed under SST conditions may present several advantages in teasing apart the vocal learning process. Arguably, all birds could accurately encode the template with individual differences in template decay giving rise to a range of imitative abilities. However, the template is thought to be quite durable once encoded [24]. Therefore, much of the individual variability in imitativeness we attribute to genetic or epigenetic differences in the fidelity of encoding. Genetic contributions to song learning in an aviary setting may be overwhelmed by social influences [25] and protracted operant training may underestimate the range of imitative ability. This SST paradigm should prove valuable for teasing apart genetic contributions to song learning that may be evolutionarily conserved and applicable to human speech and language acquisition [2,26].
